# Viral‐Mediated Connexin 26 Expression Combined with Dexamethasone Rescues Hearing in a Conditional *Gjb2* Null Mice Model

**DOI:** 10.1002/advs.202406510

**Published:** 2024-12-30

**Authors:** Xiaohui Wang, Li Zhang, Sen Chen, Le Xie, Yue Qiu, Chenyang Kong, Ge Yin, Weijia Kong, Yu Sun

**Affiliations:** ^1^ Department of Otorhinolaryngology Union Hospital Tongji Medical College Huazhong University of Science and Technology Wuhan 430022 China; ^2^ Institute of Otorhinolaryngology Tongji Medical College Huazhong University of Science and Technology Wuhan 430022 China; ^3^ Hubei Province Key Laboratory of Oral and Maxillofacial Development and Regeneration Wuhan 430022 China

**Keywords:** AAV2.7m8, dexamethasone, gene therapy, GJB2, hearing loss

## Abstract

*GJB2* encodes connexin 26 (Cx26), the most commonly mutated gene causing hereditary non‐syndromic hearing loss. Cx26 is mainly expressed in supporting cells (SCs) and fibrocytes in the mammalian cochlea. Gene therapy is currently considered the most promising strategy for eradicating genetic diseases. However, there have been no significant effects of gene therapy for *GJB2* gene mutation‐associated deafness because deficiency of Cx26 leads to expanded sensory epithelial damage. In this study, the AAV2.7m8 serotype combined with the gfaABC1D promoter targeted infection of SCs is identified. It is found that *Gjb2* gene replacement therapy in wild‐type mice results in sensory hair cells (HCs) deficits, excessive inflammatory responses, and hearing loss. This may be one of the key factors contributing to the hardship of *GJB2* gene replacement therapy. Dexamethasone (DEX) shows promising results in inhibiting macrophage recruitment, with a protective effect against HC damage. Further, the combination of AAV2.7m8‐*Gjb2* with DEX shows a synergistic effect and enhances the gene therapy effect in a conditional Cx26 null mice model. These results indicate that the combination of gene therapy and medication will provide a new strategy for the treatment of hereditary deafness associated with *GJB2* defects.

## Graphical abstract

AAV2.7m8 serotype combined with the gfaABC1D promoter targets infection of supporting cells (SCs). AAV2.7m8‐gfaABC1D‐*Gjb2* administration to mice results in excessive immune responses. The combination of AAV2.7m8‐gfaABC1D‐*Gjb2* with dexamethasone (DEX) shows a synergistic effect and enhances the gene therapy effect in a conditional Cx26 null mice model.

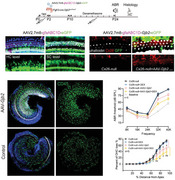

## 1 Introduction

Hearing loss (HL) is one of the most common sensory deficits in humans. Approximately 1 out of 500 infants are found to be affected by congenital HL and genetic mutations are responsible for 50–60% of these cases.^[^ ^1^ ^]^ Currently, the main treatment options for hereditary HL are hearing aids and cochlear implants, depending on the severity and type of HL. However, both of these treatments have limitations. Hearing aids are only suitable for patients with moderate to severe HL, while cochlear implants require the patient to have relatively normal auditory nerve function although they can work on totally deaf patients. To restore hearing with a more natural quality, researchers are exploring different approaches such as gene therapy and hair cell (HCs) regeneration.^[^ ^2^, ^3^, ^4^, ^5^, ^6^, ^7^ ^]^ Gene therapy, in particular, is considered the most promising approach for hereditary HL. It involves introducing a target foreign gene or gene regulatory element into target cells to replace or repair the mutated genes.^[^ ^8^, ^9^ ^]^ Significant progress has been made in inner ear gene therapy in recent years. The first successful restoration of hearing achieved through gene therapy was in *Vglut3* knockout mice.^[^ ^10^ ^]^ Subsequent studies on gene replacement therapy for hereditary deafness have emerged, targeting genes such as *Tmc1*, *Otof*, *Kcne1*, and G*jb6*.^[^ ^11^, ^12^, ^13^, ^14^, ^15^ ^]^ Gene therapy in the inner ear has also shown promising results in humans. Recent single‐arm clinical research reported that hearing and speech perception improved significantly in children with autosomal recessive deafness 9 with AAV1‐*hOTOF* administration.^[^ ^16^, ^17^ ^]^ However, there has been no great breakthrough in deafness caused by *GJB2* mutation, which is the most commonly mutated gene and accounts for up to 50% of hereditary HL.

The *GJB2* gene is responsible for the production of Connexin 26 (Cx26), which is primarily expressed in the supporting cells (SCs) membrane of the inner ear. Cx26 is widely expressed in the mammals’ inner ear, forming gap junctions between adjacent SCs or fibrocytes in the cochlea. Gap junctions play a critical role in intercellular communication by allowing the diffusion of ions, metabolites ≤1.5kDa, and small signaling molecules.^[^ ^18^, ^19^ ^]^ *GJB2* is highly conserved in humans and pathogenic variants usually result in the absence of one or several transmembrane segments and intervening loops in Cx26, which is associated with HL.^[^ ^19^, ^20^, ^21^ ^]^ In humans, *GJB2* mutations can cause profound or late‐onset progressive HL, with sensory epithelial damage being a recognized cause of deafness.^[^ ^22^, ^23^, ^24^ ^]^ Studies on Cx26 conditional knockout mouse models have shown degeneration of out hair cells (OHCs) and SCs at postnatal (P) day 14–15, which triggered secondary degeneration of spiral ganglion neurons at P30.^[^ ^25^, ^26^, ^27^, ^28^ ^]^ The different Cx26 null mice models demonstrated a highly consistent timing of cell death,^[^ ^29^ ^]^ indicating the existence of an optimal time window for the gene therapy of hereditary HL caused by *GJB2* mutations. Besides, previous studies have also shown that ectopic expression of Cx26 can lead to sensory epithelial damage. Therefore, identifying the optimal treatment time window and selecting vectors that can effectively infect target cells are crucial for gene therapy of *GJB2*‐related HL. In this study, we modified the AAV2.7m8 virus, which exhibits high infecting efficiency in SCs but relatively low expression in HCs. The modified AAV2.7m8 virus is a promising vector for gene therapy in Cx26 null mice.

Previous studies have shown that AAV can trigger immune responses in the host organisms,^[^ ^30^, ^31^, ^32^ ^]^ which may be another significant challenge to the efficacy and safety of gene therapy. The inner ear has been confirmed to have abundant macrophages, which account for ≈80–95% of immune cells.^[^ ^33^, ^34^ ^]^ A recent study showed that injection of AAV2.7m8‐tdTomato into the inner ear via the posterior semicircular canal increased macrophage presence in the inner ear.^[^ ^35^ ^]^ Glucocorticoids (GCs) are commonly used as immune regulators in clinics and have been widely used in inner ear diseases, including sudden sensorineural hearing loss (SSNHL), noise‐induced hearing loss (NIHL), ototoxic drug‐induced hearing loss, and Meniere's disease.^[^ ^36^, ^37^, ^38^, ^39^, ^40^, ^41^ ^]^ Dexamethasone (DEX), the most commonly used GC in clinical practice, has been proven to have a good clinical effect on various types of HL and is used to preserve residual hearing in cochlear implant surgery.^[^ ^42^, ^43^, ^44^ ^]^ Our previous study has demonstrated that DEX can partially rescue auditory function and protect the cochlear sensory epithelial in a longitudinally Cx26‐null model, probably through the regulation of immune responses in the inner ear.^[^ ^29^ ^]^ In this study, we observed that AAV2.7m8‐gfaABC1D‐*Gjb2* administration to wild‐type (WT) mice resulted in excessive immune responses, leading to OHC loss and HL. The administration of DEX was able to inhibit the immune responses, thereby preventing OHC damage and HL. Moreover, in a conditional Cx26 knockout mouse model, injection of AAV2.7m8‐gfaABC1D‐*Gjb2* into the inner ear followed by DEX significantly enhanced the protective effect of gene therapy.

## 2 Results

### 2.1 AAV2.7m8 Transduces SCs with High Efficiency and Specificity

**Figure 1:**
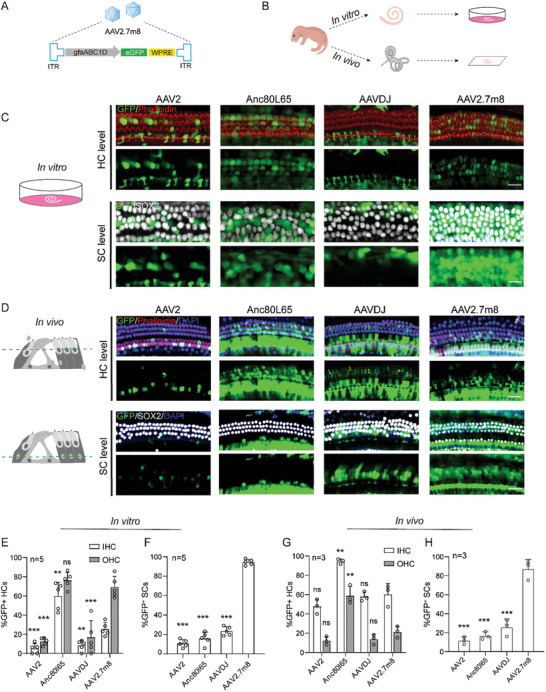
AAV‐2.7m8‐ gfaABC1D‐eGFP efficiently transduced cochlear SCs. A) Schematics of the AAV‐2.7m8‐ gfaABC1D‐eGFP genome organization. B) Process diagram of AAVs in vivo and in vitro infection of the inner ear. AAVs serotypes at the same dose (5 × 10^9^ genome‐containing (particles) (GCs) per ear). Cochleae were harvested at P21 after microinjection with 1.5 µL of AAV stock solution in one ear at P2. C,D) Representative images of eGFP fluorescence (green), Phalloidin (red), DAPI (blue), and Sox2(white) staining in the middle turns of cochleae infected with different AAVs serotypes in organotypic cochlear explants (C) and in vivo (D), respectively. Scale bar, 30 µm. (E‐H) Percentage of eGFP‐positive HCs and SCs per 100 µm corresponding to C‐D. Data are shown as mean ± SEM. Significance tests were performed between AAV‐2.7m8 and other AAV serotypes. ^*^ *p* < 0.05, ^**^ *p* < 0.01, and ^***^ *p* < 0.001. ns: non‐significant. N = 3–5 in each group. IHCs, inner hair cells; OHCs, outer hair cells; SCs, supporting cells.

First, it is necessary to find an AAV vector that can be efficiently targeted to infect SCs, as ectopic expression of Cx26 can cause cellular damage. Previous reports have found that synthetic AAV2/AAV2.7m8 together with a CAG (CMV immediate‐early enhancer chicken β‐actin) promoter efficiently expresses eGFP in HCs and inner pillar cells (IPCs) when injected at early postnatal stages.^[^ ^45^ ^]^ It has been shown that the glial fibrillary acidic protein (GFAP) promoter is mostly restricted in certain subpopulations of SCs.^[^ ^46^ ^]^ GFAP activity is observed in all SCs in the early postnatal period, with a gradient of intensity decreasing from basal to apical.^[^ ^47^ ^]^ AAV9‐PHP.B‐Gfap‐eGFP driving exogenous gene expression in SCs of the cochlea.^[^ ^48^ ^]^ Previous studies optimized the 2.2‐kb GFAP promoter into a novel 681‐bp GFAP promoter, gfaABC1D, with an expression pattern essentially identical to that of the gfa2 promoter, but with approximately two fold higher activity.^[^ ^49^ ^]^ Based on this, we generated a gene therapy vector that encapsulates the gfaABC1D promoter, target gene and/or enhanced green fluorescent protein (eGFP), and WPRE in a synthetic AAV2/AAV2.7m8 capsule: AAV2/AAV2.7m8‐gfaABC1D‐eGFP‐WPRE (abbreviated as: AAV2.7m8‐eGFP) (**Figure** **1**A).

To determine AAV serotypes with the highest viral transduction rate in cochlear SCs, several conventional AAVs were used to package an AAV reporter genome that expresses eGFP from the constitutive CMV promoter. As previously reported for broad‐spectrum promoters, the AAV‐2.7m8‐CMV mainly infected HCs (53.5%–82.1%), and some SCs (55.3%–77.1%) (Figure S1A–C, Supporting Information). We also tried the GFAP and hSyn promoter, however it only infected a small number of HCs (data not shown). Therefore, we directly compared the infection efficiency of AAV2.7m8‐gfaABC1D and different AAVs on SCs. AAV2‐eGFP, AAVDJ‐eGFP, Anc80l65‐eGFP, as well as AAV2.7m8‐eGFP at equal doses (5.0 × 10^9^ genome‐containing (particles) (GCs) per ear) were infected in the cochlear explant culture (in vitro) or injected into the inner ear of WT mice via the round window membrane (RWM) (in vivo) at P2 (Figure 1B). In organotypic cochlear explants of mice, Anc80l65 efficiently transfects HCs with efficiencies of 60.0%–76.4%, whereas infection efficiency was low in SCs. AAV2.7m8‐eGFP was expressed in 69.3% of OHCs, and AAV2.7m8‐eGFP expression was seen in almost all SOX2^+^ SC types (Figure 1C,E,F). This is in contrast to the in vivo infection of AAV2.7m8, where OHC infection was very inefficient at 21.4% efficiency (Figure 1D,G). Next, we evaluated the transfection efficiency of AAVs injected in *vivo*. As shown in Figure 1D, AAV2‐eGFP primarily transduced IHCs, while SCs were rarely transduced with low efficiencies of 11.7%. AAVDJ and Anc80L65 mainly infected HC at moderate efficiencies but the transduction efficiency of SCs was as low as 5.5% and 33.3%, respectively. Nevertheless, AAV2.7m8‐eGFP could transduce several kinds of SCs with a high transduction efficiency of 91.1% and minor amounts of OHCs with efficiencies of <21.4%.

### 2.2 AAV2.7m8‐gfaABC1D‐eGFP Efficiently Transduces Different Types of SCs In Vivo

**Figure 2:**
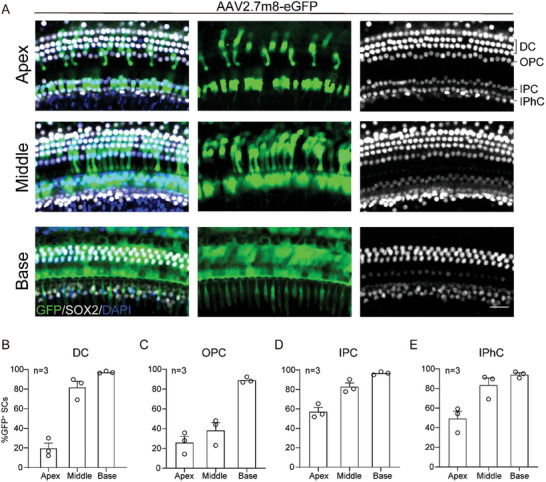
AAV2.7m8‐gfaABC1D‐eGFP efficiently transduces different types of SCs in vivo. A) Representative images of supporting cells in apical, middle, and basal turns from the AAV2.7m8‐gfaABC1D‐eGFP (GFP, green) injected at a dose of 5 × 10^9^ genome‐containing (particles) (GCs) per ear of the mice at P2. White: Sox2; Green: GFP. Blue: DAPI. Scale bar, 30 µm. B–E) Infection efficiencies of AAV2.7m8‐gfaABC1D‐eGFP in DCs, OPCs, IPCs, and IPhC, respectively. Data are shown as mean ± SEM. N = 3 in each group. DCs, Deiters cells; OPCs, outer pillar cells; IPCs: outer pillar cells; IPhC: inner phalanges cells.

With injecting a dose of 5 × 10^9^ genome‐containing (particles) (GCs) per ear of the mice at P2 and harvested at P24, fluorescence microscope experiments revealed that targeting efficiencies of AAV2.7m8‐eGFP in cochlear SCs at the apical, middle, and basal regions (**Figure** **2**A). Cochlear SCs contain different cell types: Hensen's cells (HeCs), Deiters cells (DCs), pillar cells (PCs), and inner phalangeal cells (IPhCs). The DCs targeting efficiencies at the apical, middle, and basal regions were ≈20%, 81%, and 96%, respectively (Figure 2B). The outer pillar cells (OPCs) targeting efficiencies were ≈22%, 38%, and 90%, respectively (Figure 2C). The IPCs targeting efficiencies were ≈56%, 82%, and 90%, respectively (Figure 2D). The IPhCs targeting efficiencies at the apical, middle, and basal regions were ≈48%, 85%, and 93%, respectively (Figure 2E). The infection efficiency of AAV2.7m8‐gfaABC1D‐eGFP for SCs showed a decreasing trend from base‐to‐apex turn sequentially, which was consistent with the previously described pattern of GFAP expression in the inner ear.^[^ ^47^ ^]^ In short, our results suggest that AAV2.7m8‐gfaABC1D‐eGFP can relatively specifically infect SCs with lower expression in HCs.

### 2.3 AAV2.7m8‐gfaABC1D‐eGFP is Safe for the Mouse Inner Ear

**Figure 3:**
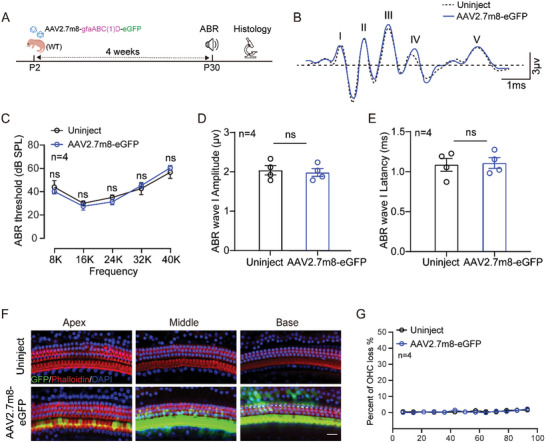
AAV2.7m8‐gfaABC1D‐eGFP is safe for the mouse inner ear. A) Diagram showing the administration mode of the AAV2.7m8‐gfaABC1D‐eGFP injection. WT mice were injected AAV2.7m8‐gfaABC1D‐eGFP at a dose of 5.0 × 10^9^ genome‐containing (particles) (GCs) per ear through RWM at P2. The mice were tested for ABR and sacrificed at P30. B) ABR waveforms were recorded in uninjected and AAV2.7m8‐eGFP injected mice. The ABR was evoked by 24 kHz tone bursts. C) Hearing thresholds of the uninjected and AAV2.7m8‐eGFP injected group at different frequencies. Data are shown as mean ± SEM. ns: non‐significant. N = 4 in each group. D,E) Statistics on the amplitude and latency of ABR wave I from (B) in uninjected and AAV2.7m8‐eGFP injected mice. Data are shown as mean ± SEM. ns: non‐significant. N = 4 in each group. F) Representative images of hair cells in apical, middle, and basal turns from the uninjected and AAV2.7m8‐eGFP injected mice at P30. Green, GFP fluorescence; Red: Phalliodin, Blue: DAPI. Scale bars = 30 µm. G) Quantification of HC loss at specific cochlear locations in the uninjected and AAV2.7m8‐eGFP injected groups. Mice injected with AAV2.7m8‐eGFP did not induce hair cell death compared to the uninjected group. Data are shown as mean ± SEM. N = 4 in each group.

We further evaluated the inner ear toxicity of AAV2.7m8‐eGFP. Mice were administered AAV2.7m8‐gfaABC1D‐eGFP through RWM at P2 and tested for auditory brain‐stem responses (ABRs) and inner ear epithelium viability at 4 weeks (**Figure** **3**A). ABR results showed there were no significant differences between the injected and uninjected ears in terms of ABR threshold, ABR wave I amplitude, and latency (Figure 3B–E). Immunofluorescence results showed that there was also no difference in inner ear OHC counts from apex to basal turn between the injected and uninjected groups (Figure 3F,G). SC counts were similarly unaffected in the injected group compared to the control mice (Data not shown). The above results indicated that AAV2.7m8‐gfaABC1D‐eGFP was a safe vector for gene therapy.

### 2.4 Administration of AAV2.7m8‐*Gjb2* to WT Mice Displayed HC Death and Hearing Loss, Prevented by the Use of a Low Dose of DEX

**Figure 4:**
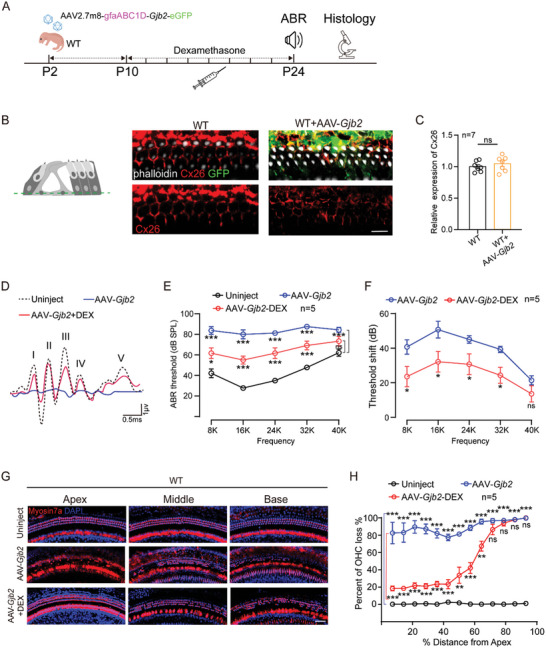
AAV2.7m8‐*Gjb2* successfully expresses Cx26 and the injection of AAV2.7m8‐*Gjb2* into wild‐type (WT) mice leads to HC death and hearing loss, a process that can be inhibited by DEX. A) Schematic overview of the experimental process of AAV2.7m8‐*Gjb2* injection and DEX administration of the WT mouse model. The mice were injected with AAV2.7m8‐gfaABC1D‐*Gjb2* (5 × 10^9^ genome‐containing (particles) (GCs) per ear) at P2. Dexamethasone was administered to mice at a dose of 3 mg kg^−1^ every two days starting at P10‐24. All of the mice were tested for auditory brainstem response (ABR) and sacrificed at P24–P25. B) Cx26 (red) and GFP (green) immunolabeling in the middle turn of the WT and WT+AAV‐*Gjb2* at a dose of 5 × 10^9^ genome‐containing (particles) (GCs) per ear of the mice at P2 respectively. White: phalloidin. Scale bars = 30 µm. C) Relative expression of Cx26 in different groups of SCs from B. Cx26‐positive cell expression rate has been used as a reference for WT mice. Data are shown as mean ± SEM. ^***^ *p* < 0.001. N = 7 in each group. D) ABR waveforms were recorded in uninjected, AAV2.7m8‐*Gjb2* and AAV2.7m8‐*Gjb2*+DEX administration mice. The mice were injected with vehicle or dexamethasone at a dose of 3 mg kg^−1^ every two days starting at P10‐24. The ABR was evoked by 24 kHz tone bursts. E) ABR thresholds of the uninjected, AAV2.7m8‐*Gjb2* and AAV2.7m8‐*Gjb2*+DEX groups at different frequencies. Data are shown as mean ± SEM. ^*^ *p* < 0.05, ^**^ *p* < 0.01, and ^***^ *p* < 0.001. N = 5 in each group. F) ABR threshold shift in AAV2.7m8‐*Gjb2* and AAV2.7m8‐*Gjb2*+DEX administration group at different frequencies. Data are shown as mean ± SEM. ^*^ *p* < 0.05, ^**^ *p* < 0.01, and ^***^ *p* < 0.001. ns: non‐significant. N = 5 in each group. G,H) Representative images and quantification of OHC loss at specific cochlear locations in the uninjected, AAV2.7m8‐*Gjb2* and AAV2.7m8‐*Gjb2*+DEX groups. Scale bars = 50 µm. Data are shown as mean ± SEM. ^*^ *p* < 0.05, ^**^ *p* < 0.01, and ^***^ *p* < 0.001. ns: non‐significant. N = 5 in each group.

Considering the specificity and safety of AAV2.7m8 in targeting SCs, we packaged the murine‐derived *Gjb2* together into the virus: AAV2/AAV2.7m8‐gfaABC1D‐*Gjb2*‐eGFP‐WPRE (abbreviated as: AAV2.7m8‐*Gjb2*). We first infected AAV‐*Gjb2* in HEK293 cells, and the result showed that Cx26 could be successfully expressed and displayed viral titer dependence (Figure S1D,E, Supporting Information). Subsequently, conditional knockdown of *Gjb2* in SCs was induced by Tamoxifen in mice P0‐P1, and AAV‐*Gjb2* was injected by RWM at P2 (**Figure** **4**A). Immunofluorescence results after 3 weeks showed that Cx26 could be successfully co‐expressed on the membranes of the SCs together with eGFP (Figure 4B,C).

And yet, we found that the operated sides of WT mice injected with AAV2.7m8‐*Gjb2* exhibited elevated ABR thresholds along with significant mortality of HCs, compared with the contralateral uninjected ears. Previous studies have shown that DEX can be used routinely as a perioperative medication in inner ear surgery to combat the immune response and can transiently enhance transgene expression.^[^ ^50^, ^51^ ^]^ Considering the minimal side effects of DEX as well as prophylactic administration, we chose to start regular subcutaneous administration of DEX in mice at P10.^[^ ^29^ ^]^ We injected 3mg of DEX subcutaneously every two days from P10‐24 in WT mice with the injection of AAV2.7m8‐*Gjb2*. At P24, the auditory function of the mice was examined by ABR. As shown in Figure 4D,F, compared to the mice injected only with AAV2.7m8‐*Gjb2*, the AAV2.7m8‐*Gjb2*+DEX group showed significantly reduced auditory threshold shifts at 8, 16, 24, and 32k, with reductions of 24.3, 30.0, 24.2, and 17.9 dB respectively (*P* < 0.05, n = 5) (Figure 4F). Then, we labeled the HCs with Myosin7a by immunofluorescence staining, to examine whether DEX could attenuate the loss of OHCs. Consistent with the reduction of auditory threshold shifts, treatment with DEX significantly prevented OHC loss in apical and middle turns (Figure 4G). Mice in the AAV2.7m8‐*Gjb2* group showed extensive OHC death throughout the entire length of the basilar membrane, with an HC mortality rate of 81.3%–99.8%, and almost no effect on IHCs (Figure S1F, Supporting Information). However, administration of DEX alleviated 28.3%‐67.9% of OHC death in the range of 60% from the top of the apex, with the region beyond the base turn showing no significant salvage effect (Figure 4H). These results indicated that DEX can prevent OHC loss and alleviate auditory threshold shifts caused by the administration of AAV2.7m8‐*Gjb2* in WT mice.

### 2.5 Inner Ears of the AAV2.7m8‐*Gjb2* Injection Group Presented Activation and Recruitment of CD45^+^ Macrophages

**Figure 5:**
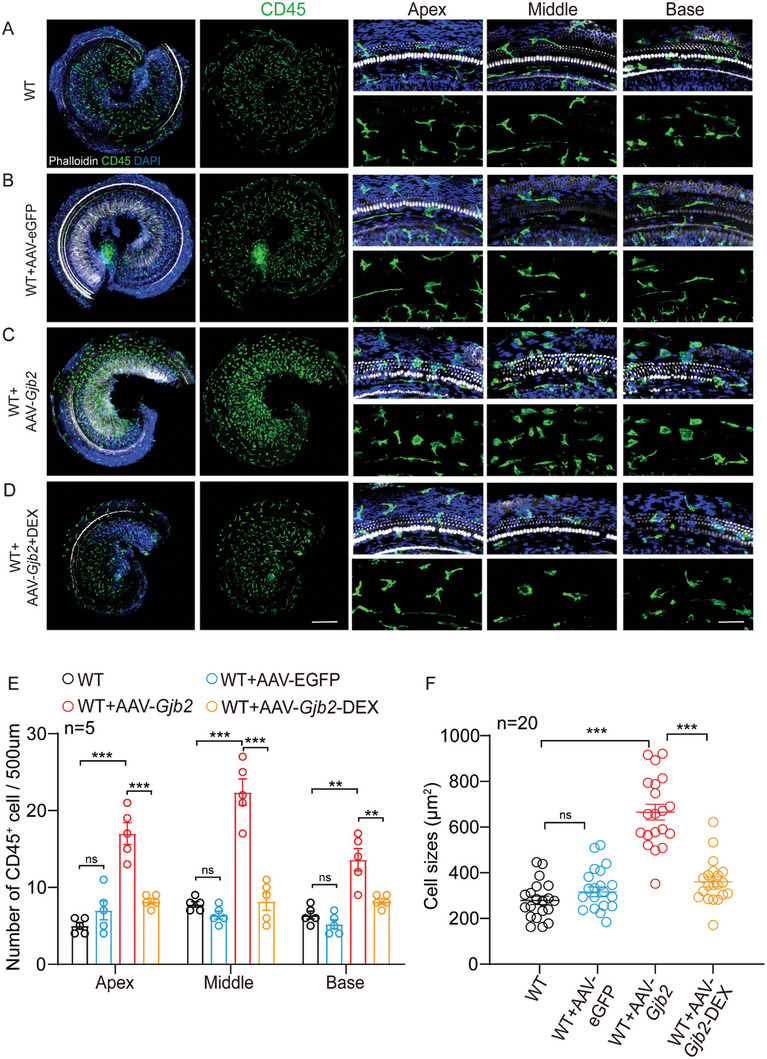
Distribution of CD45^+^ macrophages in AAV2.7m8‐*Gjb2* injection group. Mice were injected with AAV2.7m8‐gfaABC1D‐eGFP or AAV2.7m8‐gfaABC1D‐*Gjb2* (5 × 10^9^ genome‐containing (particles) (GCs) at P2 and the cochlea was harvested at P24. Beginning at P10‐24, mice were administered dexamethasone at a dose of 3 mg kg^−1^ once every two days. A–D) Immunofluorescent staining of CD45^+^ macrophages (green) in the apical, middle, and basal turns of different groups. Blue: DAPI. White: F‐actin. Scale bars represent 200 and 30 µm, respectively. E,F) Comparison of the numbers and the macrophage size of CD45^+^ cells in the different groups. Data are shown as mean ± SEM. ^*^ *p* < 0.05, ^**^ *p* < 0.01, and ^***^ *p* < 0.001. ns: non‐significant.

It has been reported that the host immune response plays an important role in the safety and efficacy of viral‐mediated gene therapy.^[^ ^35^, ^50^, ^51^ ^]^ We inferred that the exogenous Cx26 may induce inflammatory responses in the inner ear, which could be the primary cause of death of OHC. We performed immunofluorescence detection to reveal the expression of CD45, which showed the morphology and distribution pattern of macrophages in the cochlea. In the inner ear injected AAV2.7m8‐eGFP group, cell counts showed no difference in the number of CD45^+^ cells in different regions compared to the sham‐operated control group from basal to apical turns (**Figure** **5**A,B). However, the number of CD45^+^ cells in the inner ear appeared to increase significantly in the AAV2.7m8‐*Gjb2*‐injected group, which was 2.4–3.5 fold higher than in the AAV2.7m8‐eGFP‐injected group (Figure 5C,E). The number of CD45^+^ macrophages was significantly reduced by 39.7–63.4% after administration of low‐dose DEX as previously described (Figure 5D,E).

In addition, we examined macrophage morphology, which is an important indicator of macrophage activation. In the control and AAV2.7m8‐eGFP injection groups, CD45^+^ cells showed a slender or striped shape (Figure 5A,B). However, most of the CD45^+^ cells near the cochlear damage in the AAV2.7m8‐*Gjb2* group showed enlarged cell bodies and dendritic projections, suggesting that they were activated macrophages (Figure 5C). Quantitative statistics of cell size showed that the mean size of CD45 cells in the middle of the cochlea in the AAV2.7m8‐*Gjb2* group (655.5 ± 150.5 mm^2^) was significantly larger than that in the control and AAV2.7m8‐eGFP groups (320.9 ± 95.2, 308.0 ± 83.4 mm^2^, respectively, Figure 5F). After the use of low‐dose DEX, the size of CD45^+^ cells was significantly reduced (360.6 ± 94.6 mm^2^, *p* < 0.001 compared with the AAV2.7m8‐*Gjb2* injection group), but their shape was more oriented toward a rounded protruding shape (Figure 5D,F). These results indicated that inflammation may be an essential reason for the poor efficacy of gene therapy.

### 2.6 Administration of Low‐Dose DEX Alongside AAV2.7m8‐*Gjb2* Notably Improved Hearing in a Conditional *Gjb2* Null Mice Model

**Figure 6:**
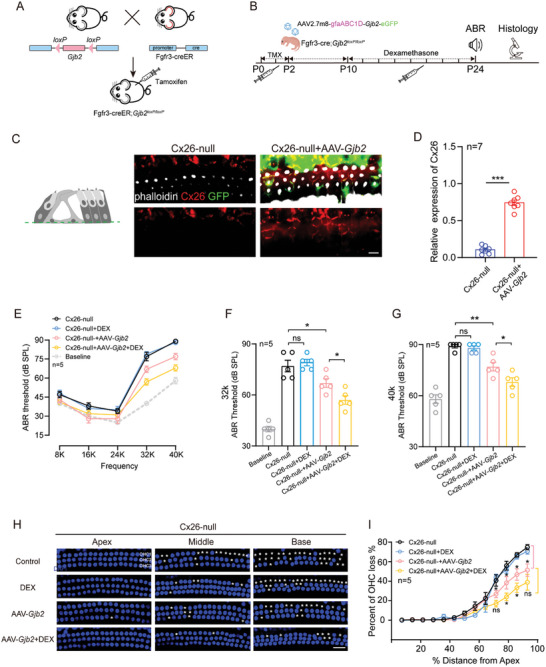
Rescue effect of the combination of AAV2.7m8‐*Gjb2* and DEX in a conditional *Gjb2* null mouse model. A) Schematic representation of the crossbreeding strategy to generate the targeted *Gjb2*‐null mouse model Fgfr3‐creER; *Gjb2* ^loxp/loxp^. B) Schematic overview of the experimental process of AAV2.7m8‐*Gjb2* injection and DEX administration of the Fgfr3‐creER; *Gjb2* ^loxp/loxp^ mouse model. The mice were injected subcutaneously with tamoxifen (TMX) at P0 and P1 and injected with AAV2.7m8‐gfaABC1D‐*Gjb2* (5 × 10^9^ genome‐containing (particles) (GCs) per ear) at P2. Dexamethasone was administered to mice at a dose of 3 mg kg^−1^ every two days starting at P10‐24. All of the mice were tested for auditory brainstem response (ABR) and sacrificed at P24–P25. C) Cx26 (red) and GFP (green) immunolabeling in the middle turn of the Cx26‐null andCx26‐null+AAV‐*Gjb2* at a dose of 5 × 10^9^ genome‐containing (particles) (GCs) per ear of the mice at P2 respectively. White: phalloidin. Scale bars = 30 µm. D) Relative expression of Cx26 in different groups of the SCs from C. Cx26‐positive cell expression rate has been used as a reference for WT mice. Data are shown as mean ± SEM. ^***^ *p* < 0.001. N = 7 in each group. E) ABR thresholds of the different groups at 8–40K frequencies. ABR baseline thresholds were derived from wide‐type (WT) mice hearing at the same age. Data are shown as mean ± SEM. N = 5 in each group. F,G) Comparison of the changes in ABR thresholds at 32 and 40K in different groups, respectively. Data are shown as mean ± SEM. ^*^ *p* < 0.05, ^**^ *p* < 0.01, and ^***^ *p* < 0.001. ns: non‐significant. N = 5 in each group. H) Representative images of hair cells in apical, middle, and basal turns from the control, DEX, AAV‐*Gjb2*, and AAV‐*Gjb2*+DEX group of the Cx26‐null mice. White asterisks indicate the absence of OHCs in the middle or basal cochlea. Scale bars = 30 µm. I) Quantification of OHC loss at specific cochlear locations in the groups from F. Data are shown as mean ± SEM. ^*^ *p* < 0.05, ^**^ *p* < 0.01, and ^***^ *p* < 0.001. ns: non‐significant. N = 5 in each group.

Gene therapy for *GJB2*‐deficient mice remains a great challenge. Next, we investigated whether AAV2.7m8‐*Gjb2* administration could rescue the hearing of Cx26 null mice with or without the treatment of DEX. First, the Fgfr3‐CreER; *Gjb2* ^loxp^/^loxp^ mice were subjected to targeted knockout of *Gjb2* in DCs and PCs by Tamoxifen (TMX) induced Cre recombinase (**Figure** **6**A). Fgfr3 is a transcription factor specific for DCs and PCs in the inner ear, and its induced targeted‐SCs‐*Gjb2* knockout mice demonstrate high‐frequency HL accompanied by loss of HCs from base to apex.^[^ ^22^ ^]^ AAV2.7m8‐*Gjb2* was injected into the cochlea of Cx26 null mice through RMW. Then, the Cx26 null mice were divided into 2 groups: AAV2.7m8‐*Gjb2* group, which was only administrated AAV2.7m8‐*Gjb2*; DEX group, which low‐dose DEX was administrated from P10‐24 every two days following the delivery of AAV2.7m8‐*Gjb2* (Figure 6B). At P24, different groups of mice (Cx26‐null group, Cx26‐null+DEX group, Cx26‐null+AAV‐*Gjb2* group, and the Cx26‐null+AAV‐*Gjb2*+DEX group) were harvested to measure the ABR and the epithelial sensory cells of the inner ear. ABR baseline thresholds were derived from WT mice hearing at the same age. Consistent with our previous results, low doses of DEX had no rescue effect on deafness in Fgfr3‐CreER; *Gjb2^loxp^/^loxp^* mice. Immunofluorescence staining showed that Cx26 could be successfully expressed in SCs of Cx26‐null mice, which could be restored to 72.9% (*P*<0.001, n = 7) of that in WT mice (Figure 6C,D). We found that the ABR thresholds of mice treated only with AAV2.7m8‐*Gjb2* were only slightly preserved at 32 and 40 K (10.0 ± 10.8 and 12.0 ± 6.5 dB, respectively) compared to the control group (Figure 6E–G). Nevertheless, administration alongside AAV2.7m8‐*Gjb2* notably improved the hearing (20.0 ± 10.8 dB at 32K and 21.0 ± 6.5 dB at 40K, respectively) of the Cx26 null mice compared to the control group.

Furthermore, we performed immunolabelling of HCs to examine the morphology of the four groups. The Cx26 null mice showed OHC loss (Figure 6H), consistent with our previous study.^[^ ^22^ ^]^ Administration of AAV2.7m8‐*Gjb2* could prevent the degeneration of OHC with or without the administration of DEX (Figure 6H,I). At 92%, 85%, 77%, and 69% distances from the apex, AAV2.7m8‐*Gjb2* reduced OHC loss by 23.5%, 20.17%, 17.6%, and 13.9% compared with the Cx26‐null mice, respectively; while AAV2.7m8‐*Gjb2*+DEX reduced loss by 35.9%, 33.7%, 32.0%, and 23.8%, respectively (Figure 6F,G). In terms of the protective effect of DEX alone on HCs of Cx26 mice injected with AAV‐*Gjb2*, it further protected 14.4% and 13.5% of OHCs at 85% and 77% from the apical turn (*P*<0.05, n = 5).

**Figure 7:**
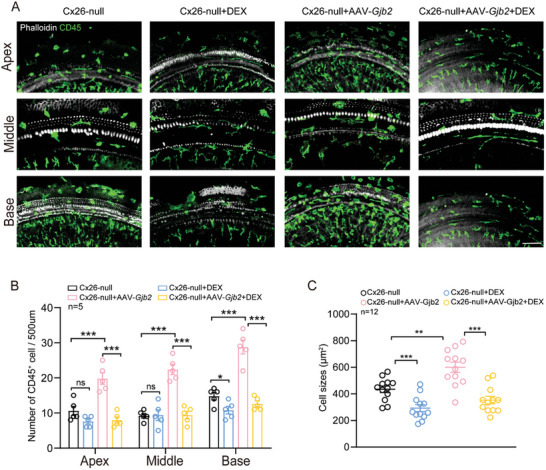
Distribution of CD45^+^ macrophages in different group. The mice were injected subcutaneously with tamoxifen (TMX) at P0 and P1 and injected with AAV2.7m8‐gfaABC1D‐*Gjb2* (5 × 10^9^ genome‐containing (particles) (GCs) per ear) at P2. Dexamethasone was administered to mice at a dose of 3 mg kg^−1^ every two days starting at P10‐24. All of the mice were tested for auditory brainstem response (ABR) and sacrificed at P24–P25. A) Immunofluorescent staining of CD45^+^ macrophages (green) in the apex, middle and base turns of different groups. White: F‐actin. Scale bars = 30 µm. B,C) Statistics of CD45^+^ cell number and macrophage size in different groups. Data are shown as mean ± SEM. ^*^ *p* < 0.05, ^**^ *p* < 0.01, and ^***^ *p* < 0.001. ns: non‐significant.

The inner ear of targeted Cx26 null mice exhibited mild activation of macrophages^[^ ^52^ ^]^ which was moderately alleviated by DEX application. Further, there were significantly more CD45^+^ macrophages (20.2 ± 2.7) and increased cell area (605.0 ± 132.5 mm^2^) in the AAV2.7m8‐*Gjb2* injected group compared to the Cx26‐null group, while DEX application attenuated the recruitment and activation of CD45^+^ cells by 53.5% in number and 42.0% in cell area in the middle turn (**Figure** **7**A–C). Activation of CD45^+^ cells showed a gradual increase from apical‐basal turns (Figure 7B). In conclusion, the above results suggest that DEX combined with AAV vectors may achieve the role of complementary gene therapy by suppressing the immune response, and the effect will be twice as effective.

## 3 Discussion

In this study, we successfully identified an AAV vector that can efficiently and specifically infect SCs with low ectopic expression in HCs. Previous studies have shown that synthetic AAV2/AAV2.7m8 with a CAG promoter can efficiently infect HCs and IPCs.^[^ ^45^ ^]^ To enhance the infection efficiency of SCs and reduce the HCs transfection, we encapsulate the gfaABC1D promoter, which has been reported to drive exogenous gene expression in SCs of the cochlea,^[^ ^48^ ^]^ into the AAV2.7m8 virus. Then, we compared the transduction efficiency of recombinant AAV2.7m8, AAV2, Anc80L65, and AAVDJ. The results showed that recombinant AAV2.7m8 exhibited higher infectivity in SCs compared to the other three viruses (Figure 1). These findings were consistent with a previous study that showed AAV2.7m8 had high transfection efficiency in IPCs.^[^ ^45^ ^]^ Additionally, the researchers discovered that the recombinant AAV2.7m8 could effectively infect DCs, OPCs, and IPhCs (Figure 2). The transfection efficiency of recombinant AAV2.7m8 for IHC and OHC in this study was relatively much lower than that reported before.^[^ ^45^ ^]^ These modifications made the recombinant AAV2.7m8 more suitable for gene therapy of Cx26 null mice. To assess the safety of the vector, we tested the hearing of WT mice injected with AAV2.7m8‐eGFP via RWM. The results showed no significant auditory threshold shift compared to the control group (Figure 3). These results indicated that the recombinant AAV2.7m8 was a suitable choice for gene therapy of Cx26 knockout mice.

Then, we injected the AAV2.7m8‐gfaABC1D‐*Gjb2* into the cochlea of WT mice and Cx26 knockout mice to confirm if the SCs could be transfected. The AAV2.7m8‐gfaABC1D‐*Gjb2* successfully infected the SCs of WT and Cx26 mice (Figure 4D). However, an interesting finding emerged when AAV2.7m8‐*Gjb2* was administered into the cochlea of WT mice. These mice showed significant HL and OHC loss in the injected ears (Figure 4E–I). This is consistent with a study by Guo et al., where injection of Anc80l65‐*Gjb2*‐Flag into the WT mice resulted in IHC loss, while Anc80l65‐eGFP did not cause significant IHC loss.^[^ ^53^ ^]^ They inferred that the difference in ectopic *GJB2* expression pattern in HCs may contribute to the observed differences. IHCs exhibit normal junctional localization of Cx26 between the cell membrane, whereas OHCs exhibit cytoplasmic localization of Cx26. In this study, OHC loss was observed after AAV2.7m8‐gfaABC1D‐*Gjb2* administration. Yu et al.’s study showed that ectopic Cx26 expression without the formation of ectopic gap junctions did not impact hearing.^[^ ^54^ ^]^ These results indicated that ectopic expression of Cx26 was not the main reason for the HL in this study. It has been reported that the immune response of the inner ear is involved in cochlear epithelial damage and the gene therapy of cochlea.^[^ ^35^, ^52^ ^]^ We suspected that immune response may be associated with the OHC loss observed in WT mice administered with AVV2.7m8‐ gfaABC1D‐*Gjb2*. Infiltrating or mature tissue macrophages are the primary immune response cells during cochlear epithelial damage.^[^ ^55^, ^56^ ^]^ In a previous study, CD45 labeling and morphological observation were used to identify macrophages/monocytes in a mouse model of noise‐induced deafness. The majority of CD45^+^ cells expressed CX3CR1, while ≈90% of CX3CR1 cells were CD68‐ and Iba‐1 positive. This discovery allowed the authors to determine that these quad‐marker positive cells were cochlear macrophages.^[^ ^57^ ^]^ The changes in CD45^+^ cells can partially represent the trend of macrophage variations. There, we examined the CD45^+^ cells in different groups and found a significant increase of CD45^+^ cells in WT with AAV2.7m8‐*Gjb2* injection group compared to the control group and WT with AAV2.7m8‐GFP injection group (Figure 5). This suggests that DEX in damaging the sensory epithelium of the cochlea and partially restoring auditory function may be related to the regulation of the immune response of the inner ear in Cx26‐deficient mouse models. Although we do not know the specific mechanism by which DEX counteracts OHC death, it suggests that it is a viable method for rescuing *Gjb2*‐related deafness based on protecting OHC death.

Our study was the first to demonstrate that DEX can effectively inhibit the macrophage‐associated immune response triggered by gene therapy in the inner ear. Our previous study showed that systemic high‐dose systemic administration of DEX could significantly reduce the recruitment of CD45^+^ cells in Cx26 null mice. This resulted in the prevention of OHC loss and preservation of hearing.^[^ ^29^ ^]^ These findings suggested that DEX could prevent OHC death and HL by suppressing immune responses in the inner ear. To further investigate this hypothesis, we administered DEX after the injection of AAV2.7m8‐gfaABC1D‐*Gjb2*. However, the previous dosage of DEX (5mg kg^−1^) was relatively high and could potentially cause a series of side effects. In this study, we opted for a safer dosage of DEX (3mg kg^−1^), which had no protective effect in Cx26 null mice. DEX was injected after the AAV2.7m8‐gfaABC1D‐*Gjb2* injection. The ABR test showed that the hearing of WT mice with DEX treatment following AAV2.7m8‐gfaABC1D‐*Gjb2* injection was improved, and the OHC loss was alleviated significantly. Additionally, the number of CD45^+^ cells decreased significantly compared to mice that only received AAV2.7m8‐gfaABC1D‐*Gjb2* administration (Figures 6 and 7). These results suggested that DEX can provide a protective effect against HL and OHC injury caused by virus injection of AAV2.7m8‐gfaABC1D‐*Gjb2* by inhibiting recruited CD45‐positive cells. It is worth noting that Fgfr3‐CreER; *Gjb2^loxP/loxP^* mice may not represent a comprehensive representation of the GJB2 knockout mouse model.

Several studies have attempted to preserve hearing in Cx26 knockout mice. Yu et al. administrated AAV2/1‐CB7‐*Gjb2* into scala media of conditional Cx26 knockout mice (Foxg1‐cCx26KO) at P0‐P1.^[^ ^54^ ^]^ The auditory function of Cx26 knockout mice did not show significant improvement although cell death in the organ of Corti and spiral ganglion neurons in the cochlea were substantially reduced. Similarly, another study delivered Anc80l65‐*GJB2*‐FLAG to adult *Sox10Cre* ^ERT2^; *Gjb2* ^flox/flox^ mice and did not observe hearing or physiological recovery.^[^ ^53^ ^]^ In our study, we observed a decrease in the hearing threshold of Cx26 knockout mice with AAV2.7m8‐gfaABC1D‐*Gjb2* injection at frequencies of 32kHz and 40kHz (Figure 6). Interestingly, the auditory thresholds at low frequencies of Cx26 null mice with AAV2.7m8‐gfaABC1D‐*Gjb2* injection did not show an increase. The discrepancy between Cx26 null mice and WT with AAV2.7m8‐gfaABC1D‐*Gjb2* injection may be attributed to the cytotoxicity resulting from the overexpression of Cx26 in SCs. Immune response has been proven to reduce the effectiveness of gene therapy in our previous results. Thus, we sought to investigate whether suppressing the immune response could enhance the efficacy of gene therapy in Cx26 null mice. The ABR test showed that the hearing of Cx26 knockout mice with AAV2.7m8‐gfaABC1D‐*Gjb2* injection followed by DEX administration further improved at frequencies of 32 and 40 kHz compared to those that only received AAV2.7m8‐gfaABC1D‐*Gjb2* injection. The improvement in hearing was consistent with a reduction in OHC loss (Figure 6). Interestingly, DEX had different rescue effects for the WT+AAV‐*Gjb2* group and the Cx26‐null+AAV‐*Gjb2* group. The former was located primarily in the apical turn of the cochlea, whereas the latter was located primarily in the middle‐basal turns. We considered possible reasons including:^[^ ^1^ ^]^ Different causes of HC death in two groups. The loss of HCs in the WT+AAV‐*Gjb2* group might be related to the abnormal overexpression of Cx26.^[^ ^53^ ^]^ In contrast, the Cx26‐null group was caused by a defect in *Gjb2* in the SCs.^[^ ^2^, ^22^ ^]^ The recovery patterns of HC in the inner ear are diverse. The mechanism of HC impairment in different regions is not clear. In animal models of NIHL, systemic administration of DEX has been shown to primarily alleviate HL in the low to medium frequency range.^[^ ^58^ ^]^ In a mouse model of cisplatin‐induced deafness, cisplatin predominantly impaired high‐frequency hearing, and DEX demonstrated great efficacy in alleviating high‐frequency hearing.^[^ ^59^ ^]^ In the recent results of AAV‐otof gene therapy for human otof gene mutations, the therapeutic effect was shown to vary depending on the pattern of hearing damage in different patients, such as patients with predominantly low‐frequency hearing damage, whose hearing recovery was mainly in the low frequency, and vice versa, and patients with full‐frequency hearing damage, who had a balanced recovery.^[^ ^15^, ^16^ ^]^ However, the clear mechanism of death requires further investigation. The number and size of CD45‐positive cells decreased significantly. In conclusion, our study provides evidence that low‐dose DEX could enhance the effectiveness of gene therapy by suppressing the immune response. This suggested that DEX may be a promising candidate for improving the efficacy of gene therapy in the inner ear.

In conclusion, our study provides evidence supporting the use of AAV2.7m8‐gfaABC1D‐*Gjb2* in combination with DEX as a potential strategy for rescuing the hearing of Cx26 null mice and minimizing the side effects of each other, providing a new therapeutic strategy for the treatment of hereditary hearing loss.

## 4 Experimental Section

### Virus Preparation

The cut‐off cDNAs of eGfp and Mus musculus *Gjb2* gene were inserted into the AAV plasmid containing the gfaABC1D promoter and the woodchuck hepatitis virus post‐transcriptional regulatory element (WPRE) cassette to form the virus of AAV2.7m8‐gfaABC1D‐eGfp and AAV2.7m8‐gfaABC1D*‐Gjb2‐*eGfp. AAV2, Anc80l65, and AAVDJ control viruses packaged with the CMV promoter to form the AAV2‐CMV‐eGfp, Anc80l65‐CMV‐eGfp, and AAVDJ‐CMV‐eGfp. The AAV was prepared using triple‐plasmid transfection of HEK293T cells. The main pAAV plasmid contained AAV2 ITRs. Separate Rep/Cap plasmid and the helper Plasmid provided components of the viral replication machinery and the capsid proteins of selected AAV serotypes. After HEK293 cell lysis, Viral particles were purified by CsCl gradient ultracentrifugation. And rAAVs were titered by quantitative polymerase chain reaction (qPCR). The viral tools were all packaged by BrainVTA (BrainVTA Co., Ltd, Wuhan, China). The virus aliquots (10µl each, in phosphate buffered solution (PBS) were stored in a −80 °C freezer and thawed at room temperature before the administration.

### Culture of Cochlear Explants

The preparation of the tail collagen gel matrix has been described in detail in the previous studies.^[^ ^52^ ^]^ Briefly, the collagen gel matrix was prepared with 9 parts of rat tail collagen (Type 1–4236, BD Biosciences), 1 part of 10× Basal Medium Eagle (BME) solution (B9638, Sigma–Aldrich), and 1 part of 2% sodium carbonate (P1110, Solarbio). The collagen gel matrix was dropped on the culture dish, and 1.3 mL serum‐free medium was added after the gel became solid. Wild‐type C57BL/6 mice were sacrificed at P3, and the cochleae were quickly separated from the temporal bone. Afterward, the cochlear explants were carefully isolated and placed on the collagen gel matrix. Then, the culture dish was transferred to an incubator at 37 °C with 5% CO_2_ overnight before each treatment. On the second day, 5 × 10^10^ GC AAV was added to the medium and incubated for 48 h, then the medium was changed and incubation continued for 24h. Cochlear explants after AAV treatment were washed in PBS and then fixed for immunofluorescence.

### Virus Injection Via RWM

The vectors were injected into the left ear of the WT or Cx26 null mice at P2. The mice were anesthetized by placing them on ice until they stopped moving. Then the anesthetized mice were placed under the microscope during the whole surgery. The area around the ear was cleaned and sterilized with 75% ethanol to reduce the risk of infection. The otic bulla was first exposed by incising the skin behind the left ear. Then the round window membrane (RWM) was located by its anatomical relation to the stapedius artery. The tip of the glass pipette, made on a horizontal pipette puller (Narishige PC‐100 Pipette Puller Launched, Digitimer, UK), was advanced to penetrate the RWM to inject 1.5µl of virus fluid by using a Picospritzer III pressure microinjection system (WPI, Sarasota, FL, USA). Fast green dye (Sigma–Aldrich, St. Louis, MO, USA) was used to help visually monitor and confirm the fluid injection. The mice were moved to a 37 °C heating pad for recovery after surgeries. After recovering, the mice were returned to the animal housing facility for normal feeding. Any damaged cochlea was excluded from subsequent studies. Injection success rates ranged from 70% to 90%.

### Mouse Model

Animal care and all experimental procedures followed the policies of the Committee on Animal Research of Tongji Medical College, Huazhong University of Science and Technology. Fgfr3‐CreER; *Gjb2* ^loxp^/^loxp^ mice were chosen as experimental mice, which were obtained through crossbreeding *Gjb2* ^loxp^/^loxp^ mice and Fgfr3iCreERT2 mice. Tamoxifen (TMX, T5648‐1G, Sigma–Aldrich) was injected subcutaneously once a day at P0 and P1 (the total dose was 1.5 mg/10 g body weight,). A targeted DCs and PCs Cx26‐null mouse model was established.^[^ ^22^ ^]^ CBA/CaJ WT mice were used as the control group. All experimental mice were raised in the specific‐pathogen‐free Experimental Animal Center of Huazhong University of Science and Technology. The mice were housed at 22 ± 1 °C under a standard 12 h light/dark cycle. The animals were free to water and had a regular mouse diet. The assigned approval number of the ethical approval for animals is [20211IACUC Number: 3638].

### DEX Treatment In Vivo

To test the protective effect of DEX on the inner ear of AAV‐injected mice, mice were given regular systemic administration of DEX at P10‐P24 after the injection of the virus. Mice in the DEX intervention group were injected subcutaneously with DEX every two days for two weeks. Two concentrations of DEX (3 and 5 mg kg^−1^) were tested in our preliminary study, based on the literature.^[^ ^60^, ^61^ ^]^ The DEX was diluted in sterile saline at 0.5 mg mL^−1^ and a final dose of 3 mg kg^−1^ of body weight was used in this study. The control group or Cx26‐null group received the same volume of saline.

### Measurement of the Auditory Brainstem Responses

The hearing thresholds of mice were measured by ABR at P24. The details for ABR test protocols have been described in the published paper.^[^ ^62^ ^]^ Briefly, Mice were deeply anesthetized with a mixture of ketamine (120 mg kg^−1^) and chlorpromazine (20 mg kg^−1^). The recording electrode, reference electrode, and grounding electrode were inserted under the skin of the skull, tested ear, and contralateral ear respectively. The hearing thresholds were tested at 4, 8, 16, 24, 32, and 40 kHz by tone burst stimuli, which were generated by the TDT system (RZ6, Tucker–Davis Tech., Alachua, FL, USA). The loudspeaker (MF‐1, Tucker–Davis Tech.) was placed 10 cm away from the tested ear with another ear plugged.

### Immunofluorescence

The mice were sacrificed at P24 and cochleae were harvested. The cochleae were fixed with 4% paraformaldehyde for 2 h and decalcification with 10% sodium EDTA solution for 24 h. The dissected cochleae were blocked with 5% Bovine Serum Albumin at room temperature for 1 h after being rinsed with 0.1% Tween‐20 in PBS (PBST) three times. Then, they were incubated with primary antibodies: polyclonal mouse anti‐Cx26 (1:200 dilution, Cat#33‐5800, Invitrogen), polyclonal rabbit anti‐myosin7a (1:500 dilution, Cat# 25–6790, Proteus Bio‐Sciences), polyclonal rabbit anti‐GFP (1:200 dilution, Cat#598, Medical & Biological Laboratories, MBL), anti‐CD45 polyclonal antibody (1:100 dilution, AF114, R&D Systems, Minneapolis, MN, United States). The samples were washed three times with PBST and then incubated with secondary fluorescent antibodies (1:200 dilution, Antgene, China) for 2 h at room temperature. Nuclei and F‐actin staining were labeled with DAPI and phalloidin (P5282; Sigma, USA) for 10 min. The samples were visualized under a laser scanning confocal microscope (Nikon, Tokyo, Japan).

### Protein Extraction and Western Blotting

Protein extraction was performed 48 h after HEK293T transfection of AAV2.7m8‐gfaABC1D*‐Gjb2‐*eGfp. Transfection titers were sequentially 5 × 10^7^ GC/ml (+), 5 × 10^9^ GC/ml (++), and 5 × 10^11^ GC/ml (+++). Cells were collected and homogenized in radio immunoprecipitation assay (RIPA) lysis buffer (Cat# P0013B, Beyotime Institute of Biotechnology) and sonicated. Protein was quantified using the bicinchoninic acid protein assay kit (Cat# P0012S, Beyotime Institute of Biotechnology). Samples were loaded into 8%–12% sodium dodecyl sulfate‐polyacrylamide gel electrophoresis (SDS‐PAGE) gels (Cat# P0012A, Beyotime Institute of Biotechnology) and then transferred to polyvinylidene difluoride membranes. Membranes were incubated in a blocking buffer consisting of Tris‐buffered saline with 0.1% Tween 20 containing 5% milk for 1 h at room temperature with a subsequent overnight incubation at 4 °C with the primary antibodies. The following antibodies were used: anti‐connexin 26 (1:1000 dilution; Cat# 512800, Invitrogen) and anti‐GAPDH (1:5000 dilution; Cat# ANT425, Antgene Biotechnology Company Ltd). The membranes were then washed and probed with horseradish peroxidase‐conjugated goat anti‐rabbit secondary antibody (1:5000 dilution, Cat# ANT020, Antgene Biotechnology Company Ltd) or goat anti‐mouse secondary antibody (1:5000 dilution, Cat# ANT019, Antgene) for 1 h at room temperature. Bands were visualized using an electrochemiluminescence (ECL) reaction kit (Cat# P0018, Beyotime Institute of Biotechnology). Protein levels were measured using ImageJ software (National Institutes of Health) and normalized to GAPDH levels in the corresponding lanes.

### Statistical Analysis

All data are presented as the means ± SEMs and were graphed using GraphPad Prism (Version 8.2.1, GraphPad Software Inc). When only one factor was involved, one‐way analysis of variance (ANOVA) followed by Dunnett's multiple comparison test was used. When two factors were involved, a two‐way ANOVA multiple comparison test was used. A *p*‐value <0.05 was considered statistically significant.

## Conflict of Interest

The authors declare no conflict of interest.

## Author Contributions

X.W and L.Z. contributed equally to this work. Y.S. and W.K. conceived and designed the experiments. Material preparation, data collection, and analysis were performed by X.W., S.C., L.X., Y.Q., C.K., and G.Y. The first draft of the manuscript was written by L.Z. and X.W. All authors commented on previous versions of the manuscript. All authors read and approved the final manuscript.

## Supporting information



Supporting Information

## Data Availability

All data are available in the main text or the supplementary materials.
