# Supplementary material for: Viral‐Mediated Connexin 26 Expression Combined with Dexamethasone Rescues Hearing in a Conditional Gjb2 Null Mice Model
Source: Adv Sci (Weinh). 2024 Dec 30;12(29):2406510. doi: 10.1002/advs.202406510 (PMC12362743; doi:10.1002/advs.202406510)
Supplement: Supplementary file 1 — Supporting Information [file ADVS-12-2406510-s001.pdf]

ADVANCED SCIENCE
Open Access

Supporting Information

for *Adv. Sci.*, DOI 10.1002/advs.202406510

Viral-Mediated Connexin 26 Expression Combined with Dexamethasone Rescues Hearing in a Conditional *Gjb2* Null Mice Model

Xiaohui Wang, Li Zhang, Sen Chen, Le Xie, Yue Qiu, Chenyang Kong, Ge Yin, Weijia Kong and Yu Sun**

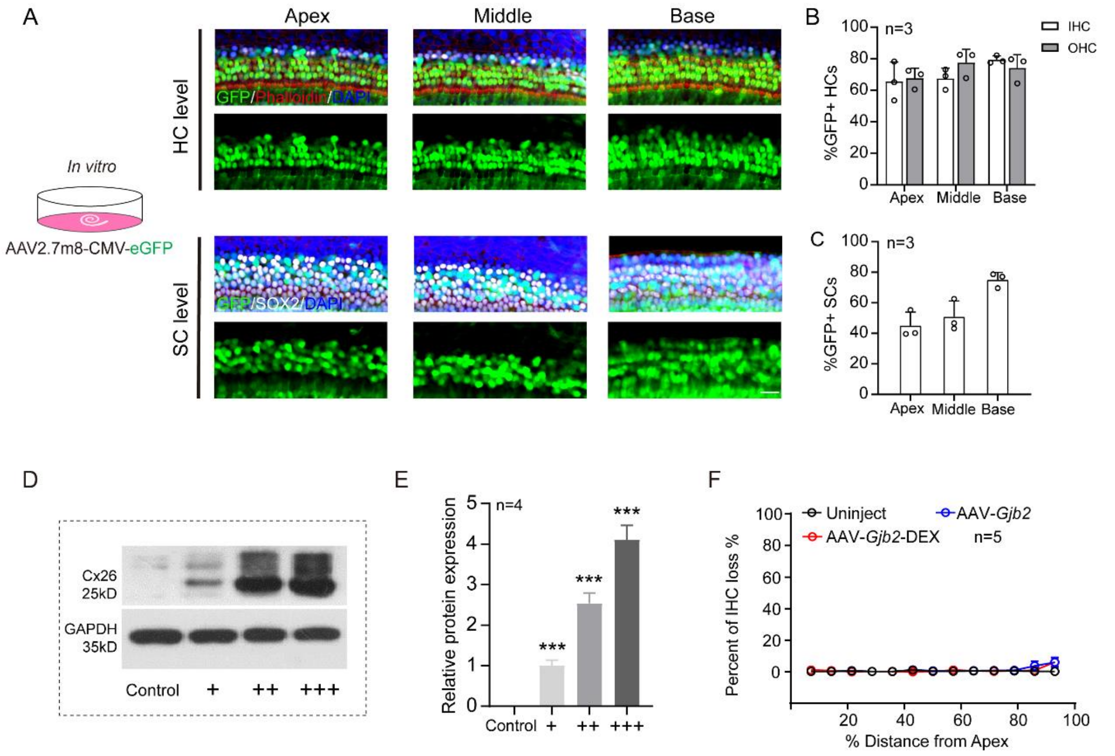

Figure S1. (A) Representative images of eGFP fluorescence (green), Phalloidin (red), DAPI (blue), and Sox2(white) staining in the apex, middle and base turns of cochleae infected with AAV2.7m8-CMV-eGFP in organotypic cochlear explants. AAVs serotypes at the same dose (5×10^9 genome-containing (particles) (GCs) per ear). Cochleae were harvested at P21 after microinjection with 1.5 μL of AAV stock solution in one ear at P2. Scale bar, 30 μm. (B, C) Percentage of eGFP-positive HCs and SCs per 100 μm corresponding to A. Data are shown as mean ± SEM. N = 3 in each group. (D, E) Western blot and histogram of Cx26 in HEK293T cells transfected with different titers of AAV2.7m8-gfaABC1D-*Gjb2*-eGfp. Transfection titers were sequentially 5×10^7 GC/ml (+), $5{\times}10^9$ GC/ml (++), and $5{\times}10^{11}$ GC/ml (+++)**.** Data are shown as mean ± SEM. ***p < 0.001. N = 4 in each group. (F) Quantification of IHC loss at specific cochlear locations in the uninjected, AAV2.7m8-*Gjb2* and AAV2.7m8-*Gjb2*+DEX

groups. N = 5 in each group.
